# Afflictive

**Published:** 1884-09

**Authors:** 


### AFFLICTIVE.

Although it cannot heal the wound, it may be some consolation to Dr. and Mrs. A. W. Harlan, of Chicago, to know that they have the deep sympathy of every member of the profession in their affliction at the loss of their oldest son, Allison Wright, who was drowned in Crystal Lake, August 19th. There is a great host of friends whose only regret is that they can do nothing but offer heartfelt condolence.

